# Systematic review on the role of the gut microbiota in tumors and their treatment

**DOI:** 10.3389/fendo.2024.1355387

**Published:** 2024-08-08

**Authors:** Ying Shi, Xiao Li, Jin Zhang

**Affiliations:** ^1^ School of Pharmacy, University College London, London, United Kingdom; ^2^ China Medical University Joint Queen’s University of Belfast, China Medical University, Shenyang, Liaoning, China; ^3^ Department of Obstetrics and Gynecology, Shengjing Hospital of China Medical University, Shenyang, Liaoning, China

**Keywords:** tumor, gut microbiota, tumorigenic effect, anti-oncogenic effect, therapy

## Abstract

Tumors present a formidable health risk with limited curability and high mortality; existing treatments face challenges in addressing the unique tumor microenvironment (hypoxia, low pH, and high permeability), necessitating the development of new therapeutic approaches. Under certain circumstances, certain bacteria, especially anaerobes or parthenogenetic anaerobes, accumulate and proliferate in the tumor environment. This phenomenon activates a series of responses in the body that ultimately produce anti-tumor effects. These bacteria can target and colonize the tumor microenvironment, promoting responses aimed at targeting and fighting tumor cells. Understanding and exploiting such interactions holds promise for innovative therapeutic strategies, potentially augmenting existing treatments and contributing to the development of more effective and targeted approaches to fighting tumors. This paper reviews the tumor-promoting mechanisms and anti-tumor effects of the digestive tract microbiome and describes bacterial therapeutic strategies for tumors, including natural and engineered anti-tumor strategies.

## 1 Introduction

**Figure 1:**
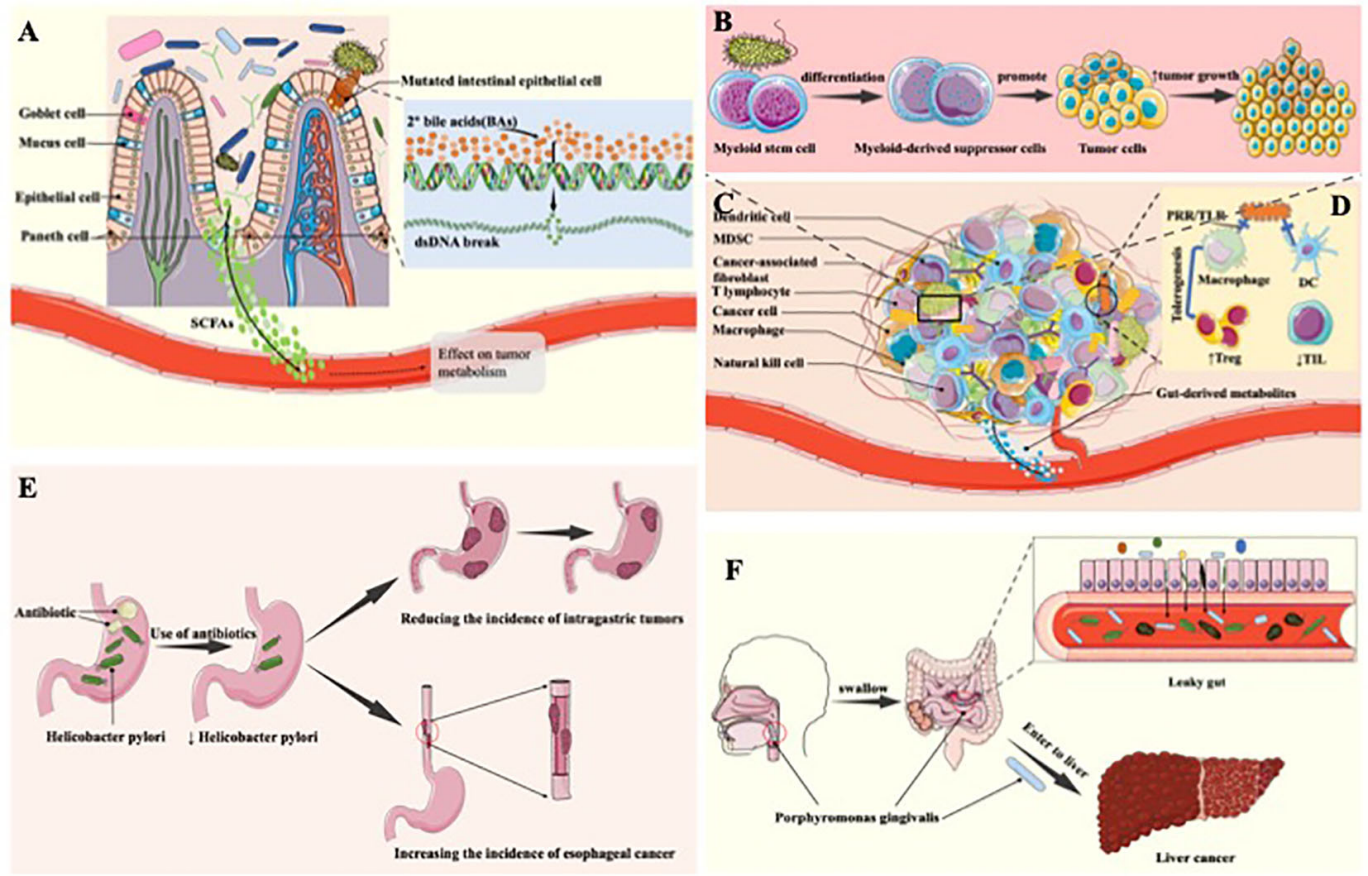
The tumorigenic effect of the gastrointestinal microbiota. **(A)** Tumor-promoting mechanism of anaerobic bacteria. Anaerobic bacteria in the gut may enzymatically convert free bile acids into secondary bile acids, which may trigger DNA damage, increase the risk of cell mutation, inhibit apoptosis, and contribute to the evolution of healthy cells into cancerous cells, thereby promoting tumour formation and progression. **(B)** The tumorigenic effect of digestive tract microbiome through induction of inflammation. The microbiome in the digestive tract triggers inflammation by releasing cytokines that stimulate the growth and proliferation of tumor cells. This inflammatory response further facilitates tumorigenesis and progression, creating a conducive environment for the development and advancement of tumors within the digestive system. **(C)** The component of TME. **(D)** The tumorigenic effect generated through change of the amount of digestive tract microbiome. In tumor patients, the digestive tract microbiome plays a role in fostering the infiltration of immunosuppressive cells by downregulating immune cells in the body. This mechanism contributes to the promotion of tumor occurrence and development, creating an environment where immunosuppression is favored and facilitating the evasion of immune responses, ultimately aiding in the progression of tumors within the digestive tract. **(E)** Antibiotic use leads to changes in digestive tract microbiome and thus tumor-promoting effects. Antibiotic use diminishes *H. pylori*, lowering the risk of gastric tumors; however, this reduction heightens the incidence of esophageal adenocarcinoma. The complex interplay highlights the dual impact of antibiotics on gastrointestinal health, underscoring the importance of considering specific cancer types when evaluating the consequences of antibiotic treatments. **(F)** Transfer of Porphyromonas gingivalis to the intestine and liver produces tumor-promoting effects. Porphyromonas gingivalis induces periodontitis in the oral cavity. Upon entering the intestinal tract, it elevates intestinal permeability, leading to “leaky gut.” This facilitates the entry of certain bacteria into the liver, potentially elevating the risk of liver cancer by creating an environment conducive to hepatic complications.

Tumors exhibit genomic instability (1), characterized by the accumulation of point mutations and structural genomic alterations throughout their development (2) (**Figure 1**). Additionally, tumors manifest a distinct tumor microenvironment (TME). Due to tumor-specific attributes, various clinical treatment methods, including radiotherapy, chemotherapy, surgery, among others, have inherent limitations, restricting their applicability and effectiveness, thus making the majority of tumors difficult to treat.

As a major “endocrine” organ of the human body, the digestive tract plays a vital role in regulating the physiological functions of the human body, such as participating in the synthesis of vitamins, amino acids and carbohydrates to maintain the normal function of the human body, preventing the invasion of pathogens and enhancing the biological barrier (3). The human gastrointestinal tract (GIT) is inhabited by various kind of microbial species, including bacteria, viruses, fungi, archaea, and protozoa (4), with bacteria being predominant and present in large numbers in the oral cavity, stomach, duodenum, jejunum and large intestine (5), but the highly acidic environment of the stomach results in low levels of bacteria in the stomach and upper small intestine. Conversely, the colon region is densely populated with an abundance of bacteria, and proximity to the colon correlates with increasing microbial load (3). The composition of the digestive tract microbiome bears a close association with tumors manifesting across various anatomical sites, including those arising within the GIT (6). A profound exploration of the impact of the digestive tract microbiome on tumorigenesis serves to elucidate the pathological mechanisms underlying malignant neoplasms. Furthermore, strategies leveraging bacterial entities for therapeutic purposes exhibit substantial promise in the domain of oncology due to the inherent tropism of bacteria towards the tumor microenvironment (7), which can overcome the shortcomings of current tumor therapies. Consequently, the investigation of the interplay between the digestive tract microbiome and tumors, particularly in the context of therapeutic interventions, stands as a prominent focal point in contemporary oncological research.

This comprehensive review aims to provide a meticulous and systematic examination of the intricate interrelationship between the digestive tract microbiome and tumors. It elucidates the mechanisms underpinning bacterial-associated strategies for tumor management and synthesizes the most recent advances in bacterial-based tumor therapeutics. The objective is to present a conceptual framework for the development of innovative strategies in tumor therapy.

## 2 The tumorigenic effect of the gastrointestinal microbiota

Microbial populations within the digestive tract including Bacteroidetes, Firmicutes, Actinobacteria, and Aspergillus (8), with this bacterial consortium encompassing not only beneficial bacteria primarily represented by Bifidobacterium and Lactobacillus, but also conditionally pathogenic organisms, notably *Escherichia coli(E. coli)*, as well as pathogenic microorganisms, prominently exemplified by Pseudomonas aeruginosa (9). In addition to the beneficial influence of beneficial bacteria on human health, both conditionally pathogenic and pathogenic bacterial organisms manifest harmful effects on the host organism. It is now widely accepted that abnormalities within the gastrointestinal microbiota may contribute to the initiation of malignant tumorigenesis (10). Simultaneously, the unique TME, characterized by internal hypoxia, low pH and increased permeability, provides an environment suitable for colonization of neoplastic lesions by a plethora of anaerobic or facultative anaerobic bacteria, take Salmonella and Escherichia (6) as examples, which in turn initiates and accelerates the process of tumorigenesis. The gastrointestinal microbiome influence tumor development through a variety of mechanisms, including releasing metabolic by-products, inducing inflammatory cascades, modulating immune responses, and altering microbial abundance and colonization sites.

### 2.1 The tumorigenic effect of digestive tract microbiota-derived metabolites

Distinct metabolites originating from digestive tract microbiome exhibit diverse impacts on tumor cells, with certain metabolites stimulating the proliferation and progression of neoplastic cells (11). The presence of bile acid hydrolases facilitates the production of free bile acids by anaerobic bacteria in the gastrointestinal tract, such as Bacteroides and Clostridium (12). Anaerobic bacteria in the intestinal environment have the potential to enzymatically convert free bile acids into secondary bile acids, thereby inducing DNA damage, increasing the probability of cellular mutations, inhibiting apoptosis, thereby promoting the transformation of healthy cells into cancer cells, and facilitating the onset and progression of tumors (13) (**Figure 1A**). Among the metabolites derived from the intestinal microbiota, short-chain fatty acids, with butyrate representing a preeminent example, have garnered extensive research attention. Investigations have unveiled that butyrate may heighten the susceptibility to tumorigenesis subsequent to genetic modifications (14). Moreover, numerous studies have revealed that butyrate amplifies the incidence of colon cancer by stimulating the proliferation of colorectal epithelial cells, resulting in the expansion of tumorigenic cell populations (15). Furthermore, certain intestinal bacteria, such as the Ruminococcaceae family within the Clostridium cluster, produce metabolites that yield β-glucuronidase, which influences estrogen levels, resulting in enhanced estrogen metabolism and free estrogen levels. Consequently, this elevation contributes to the initiation of breast tumors (11, 16, 17). It is evident that an array of GI bacterial metabolites can contribute to the initiation and advancement of tumorigenesis.

### 2.2 The tumorigenic effect of digestive tract microbiome through induction of inflammation

Recently, more and more research demonstrate that digestive tract microbiome has the potential to promote the formation and development of tumors by inducing an inflammatory response. Chronic inflammation is regarded as a significant contributing factor of the development of tumors. Cytokines and pro-inflammatory factors produced during inflammation, such as IL-1 and HMGB1 (18), which have the ability to activate cell proliferation, inhibit apoptosis, and promote angiogenesis, thus providing favorable conditions for tumor growth and dissemination (19). Arthur JC et al. manifest that the imbalance of digestive tract microbiome results in the increase of intestinal muscosal inflammation, thereby promote the development of colon cancer (20). A research article published in *Science* revealed that inflammation triggers the generation of respiratory electron acceptors, including substances like nitrate, ethanolamine, and tetrasulphite. These compounds serve as substrates for a diverse range of bacteria, including *E. coli* and *Salmonella*. Moreover, these bacteria possess significant characteristics that augment the persistence of chronic inflammation, consequently fostering the progression and proliferation of tumors (21). Furthermore, enterotoxigenic bacteroides fragilis (ETBF) generate bacteroides fragilis toxin, which involved in multiple signal transduction in colonic epithelial cells, inducing the generation of an inflammatory response that promotes the genesis and development of tumor cells (22–24) Thiele and collogues illustrated that ETBF induces myeloid stem cells to differentiate into myeloid suppressor cells, which activate pathogenic inflammatory pathways and promote colorectal cancer (CRC) development and progression (25). As a result, digestive tract microbiome induces an inflammatory response by producing cytokines that promote the growth and proliferation of tumor cells, which in turn promotes tumorigenesis and progression (**Figure 1B**).

### 2.3 The tumorigenic effect of digestive tract microbiome through modulation of the immune response

Digestive tract microbiome has the potential to promote the generation and proliferation of tumor cells through modulate human’s immune response, therefore generating the tumorigenic effect. Chamutal Gur et al. manifest that Fusobacterium nucleatum (FN) binds to the inhibitory receptor TIGIT on human natural killer cells and T cells through Fap2 protein, which inhibits the cytotoxicity of natural killer cells, thus inhibiting the anti-tumor immune function of the body and leading to tumor occurrence (26). Moreover, Robert F.Schwabe and collogues demonstrate that FN can inhibit the cytotoxicity of NK cells through TIGIT, down-regulate its inhibitory effect on tumor cells, and then promote the occurrence and development of tumors, especially colorectal adenocarcinoma tumors (27). Through an investigation involving 138 volunteers, Chen Ting and fellow researchers established a significant association between TOX protein expression and CD4^+^ T cell content within colorectal tissues. Their findings demonstrated that FN exerts a pivotal role in CRC development by reducing CD4^+^ T cell levels and suppressing TOX protein expression (28). Moreover, for individuals with Crohn’s disease exhibiting elevated FN levels leading to diminished bifidobacteria content, the susceptibility to gastrointestinal and other cancers becomes notably elevated (28). Metabolites produced by the gut microbiota, which are characterised by short-chain fatty acids like butyric acid, activate certain G protein-coupled receptors (GPCRs), especially GPR43, GPR41, GPR109A and Olfr78 (29). These bioactive compounds are vital in inducing the differentiation of nascent CD4^+^ T cells into immunosuppressive cells Tregs (30). The gut microbiota is also implicated in the progression of hepatocellular carcinoma, in addition to its impact on CRC. Research demonstrates that disruptions in gut microbiota composition caused by low-dose antibiotics or mucosal damage can drastically accelerate the advancement of hepatocellular carcinoma (31). This accelerated progression is mainly mediated by several mechanisms, including increased expression of IL-6 and activation of the nuclear factor-kappaB (NF-κB) pathway (32). Consequently, the digestive tract microbiome of tumor patients can promote the infiltration of immunosuppressive cells by downregulating the immune cells in the body, thus promoting the occurrence and development of tumors (**Figures 1C, D**).

### 2.4 The tumorigenic effect generated through change of the amount of digestive tract microbiome

The variation of the amount of digestive tract microbiome can produce tumor-promoting effect. Significant differences in the amount and composition of digestive tract flora between cancer patients and healthy populations. The gastrointestinal microbiota of healthy adults exhibits significant diversity. It is notable that Streptococcus salivarius and Streptococcus bradycosus predominate in the oral microbiome. Moving into the esophagus, one encounters Staphylococcus, Prevotella, and Veyronella populations. Stomachs contain Firmicutes, Bacteroidetes, Clostridium, Actinobacteria, Roxella and Haemophilus. As microorganisms move into the gut, diversity increases, with Proteus, Clostridium, Streptococcus and Oxalacidobacterium leading the gut environment (33). Probiotics exist in GIT possess a vital role in maintain human health (11). To be specific, an increased presence of Lactobacillus Johnsoni is linked to a decrease in genotoxicity, a reduction in pro-inflammatory factor levels, and a lower frequency of inflammatory responses. Therefore, the absence of this bacterial strain is associated with a higher susceptibility to lymphoma development in murine models (34). Furthermore, relevant reviews reported that use of antibiotic enables the imbalance of flora is digestive tract, following the influence of digestive tract tumors (35, 36). For example, *Helicobacter pylori* (*H. pylori*) has the potential to induce gastric adenocarcinoma with characteristics including lymphovascular infiltration, lymph node metastasis, and an unfavorable prognosis. These features are related to the ability of the microbe to inactivate the ARIR1A gene (37). However, Anderson and collogues manifest that the use of antibiotic leads to the decline of *H. pylori*, which reduces the incidence of gastric tumors, but increases the incidence of esophageal adenocarcinoma (38) (**Figure 1E**).

### 2.5 The tumorigenic effect generated through the transference of digestive tract microbiome

Bacteria transference also influence the generation and development of tumors. The amount of saliva swallowed by normal adults can reach 0.75–1.5L per day (39), which provides an opportunity for oral flora to flow into the digestive tract and even the intestines. At the same time, through the chewing process, the microorganisms in the mouth can be swallowed into the stomach and intestines. Porphyromonas gingivalis (P. gingivalis) causes periodontitis in the mouth, but when it enters the intestinal tract, it increases the permeability of the intestines, thus causing “leaky gut” (gut-oral axis), through which some bacteria enter the liver and increase the incidence of liver cancer (gut-liver axis) (40) (**Figure 1F**). Moreover, gut microbiome has been linked not only to gastrointestinal disorders, but also to mental illness wich can be known as Microbiota-Gut-Brain Axis (41). Metabolic, endocrine, neural and immunological pathways form the bidirectional link between the gut and the brain. These include the vagal nerve, the HPA axis, the production of bacterial metabolites, immune mediators and entero-endocrine signals (42, 43). The gut microbiota has a significant impact on neurological and psychiatric disorders such as major depressive disorder (MDD), schizophrenia (SCZ), bipolar disorder (BD), and autism spectrum disorder (ASD). Changes in the gut microbiome influence inflammation and depressive symptoms in MDD, neurotransmitter dysregulation in SCZ, immune-inflammatory activity in BD, and behavioral and sensory responses in ASD, highlighting the complexity of the gut-brain axis (41). Simultaneously, P. gingivalis can also bind to C5aR1 and TLR2 receptors on intestinal cells (44, 45), activate PI3K channel, inhibit normal apoptosis, promote intestinal inflammation, and ultimately, even promote intestinal tumourigenesis and development. Klebsiella pneumoniae coexists with the normal oral flora but carries a risk of inducing inflammatory bowel disease and potentially causing colon cancer (46). Additionally, Koji Atarashi et al. through gnotobiotic tech proved that Klebsiella pneumoniae, when isolated from the oral flora and colonized in the intestine, may act as a potent inducer of Th1 cells, thereby promoting the development of severe colon cancer through its genotoxic effects (45). It is worth mentioning that the colon of Klebsiella pneumoniae in healthy rats does not cause disease, however, when it comes to the rats used antibiotic, severe intestinal diseases may be caused, such as CRC.

### 2.6 Others

Physiological responses of the body can also promote changes in the bacterial composition of certain parts of the body that can have carcinogenic effects. For example, reflux may result in chronic oesophageal damage and promote cancer in Barrett’s oesophagus (47). Yang and colleagues revealed alterations in the oesophageal microbiota caused by reflux disease through a comparison of healthy humans’ microbiota with that of individuals who had reflux oesophagitis or Barrett’s oesophagus. The experiment found significant amounts of Streptococcus spp. in the inflamed oesophagus. The healthy oesophagus was dominated by certain bacteria, while the reflux or Barrett’s oesophagus group had a higher abundance of the Bacteroides, Aspergillus and Clostridium phylum. This is most likely due to the physiological changes caused by excess stomach acid (48). In addition, it has been demonstrated that the virulence of *H. pylori*, host genetics and environmental factors contribute to the development of gastric cancer (49). Secondly, colonization by a wide range of bacteria also promotes tumor development to some extent. Oropharyngeal or intestinal commensal bacteria (Streptococcus, Bifidobacterium, Lactobacillus, Serratia, Klebsiella, Escherichia, Pseudomonas, Neisseria, Staphylococcus and Bacillus) have been reported to be associated with gastric cancer (50–53). Additionally, *H. pylori* can modulate the microbial composition in the distal gut. Most seriously, *H. pylori* can cause low gastric acidity, which can promote the entry of acid-sensitive bacteria into the distal gastrointestinal tract, leading to changes in the colonic microbiome that may promote colon cancer development (47).

## 3 The anti-oncogenic effect of the gastrointestinal microbiota

### 3.1 The anti-oncogenic effects of digestive tract microbiota-derived metabolites

**Figure 2:**
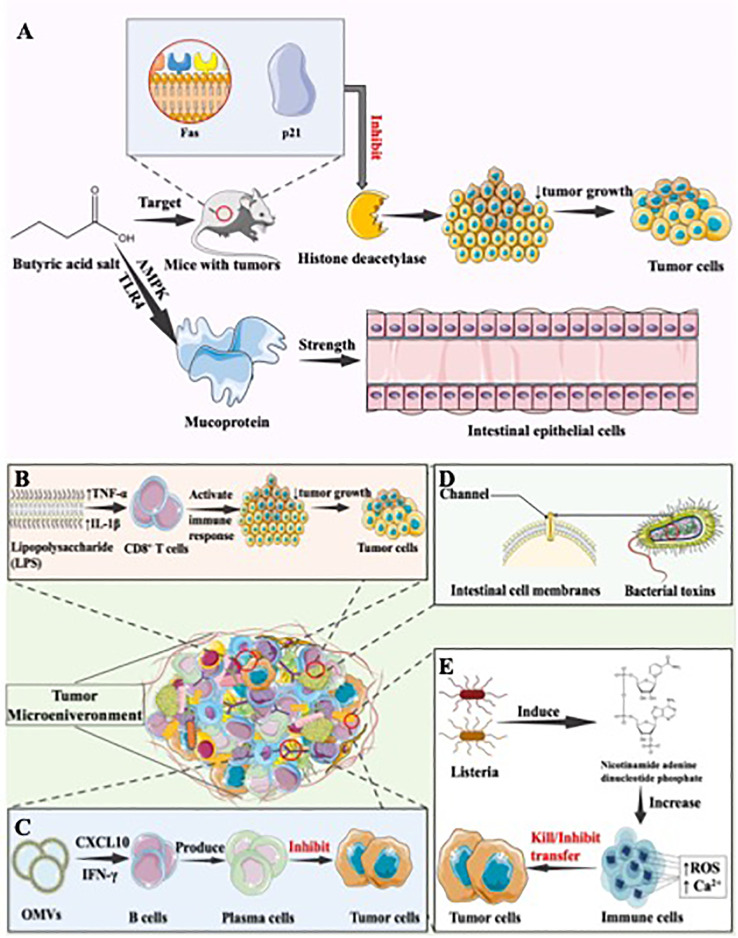
The anti-oncogenic effect of the gastrointestinal microbiota. **(A)** Antitumor mechanism of butyrate. Butyrate demonstrates anti-colon cancer effects in animal tumor models by targeting Fas and p21. It also inhibits pro-carcinogenic enzymes like histone deacetylases in the intestine. Additionally, butyrate, a short-chain fatty acid, within SCFAs, enhances the defensive role of intestinal epithelial cells by stimulating mucin secretion, further contributing to its protective influence against colon cancer, **(B)** Antitumor mechanism of Salmonella. Salmonella’s LPS component elevates the expression of TNF-α and IL-1β in the TME. This augmentation enhances the functionality of immune cells, notably CD8^+^ T cells, fostering an anti-tumor immune response with the potential to contribute to tumor suppression and immune-mediated control of cancer, **(C)** Antitumor mechanism of bacterial outer membrane vesicles. OMVs exhibit the ability to specifically target tumor tissues and prompt their rapid internalization by tumor cells. This internalization triggers the production of anti-tumor factors, such as CXCL10 and IFN-γ, and activates the human immune response. This immune stimulation not only inhibits existing tumors but also hinders the metastasis of tumors, **(D)** Antitumor mechanisms of bacterial toxins. Bacterial toxins, generated during bacterial metabolism, possess evident toxicity to the human body. These toxins have the ability to create channels in the cell membranes of eukaryotic cells, disrupting their normal barrier functions. This interference can lead to various physiological consequences, illustrating the significant impact bacterial toxins can exert on cellular processes and overall health in the context of microbial infections. **(E)** Antitumor mechanisms of inhibiting tumor metastasis. Infection with Listeria monocytogenes can activate the enzyme NADPH oxidase, which plays a critical role in the generation of ROS. Increased levels of ROS can trigger a cascade of cellular events. One of these is an increase in intracellular calcium (Ca^2+^). The increase in Ca^2+^ levels can lead to the dysfunction of the mitochondria and the activation of various apoptotic pathways, thereby leading to cell death. In the context of tumour cells, these mechanisms can lead to direct tumour cell death through the induction of oxidative stress and apoptosis, thereby effectively reducing tumour burden.

Certain metabolic byproducts of the gastrointestinal microbiota have a “biphasic” effect on tumors, which means it not only promotes the development of tumors, but also exerts anti-tumor effects. The primary gut fermentation products, short-chain fatty acids (SCFAs), play a role in regulating colonic epithelial cell growth and differentiation (54). Butyrate, which serves as a primary energy source for colonic cells, is the most extensively studied of the SCFAs (30). Butyrate can exert its anti-colon cancer effects by targeting Fas and p21 in animal tumor models, as well as by inhibiting enzymes with pro-carcinogenic activities in the intestine, such as histone deacetylases (55) (**Figure 2A**). These actions result in suppressing tumor cell proliferation and help to inhibit further CRC progression (46). Additionally, butyrate also contains the role of maintaining intestinal barrier function, which plays a significant role in the maintenance of intestinal function. Disruption of intestinal barrier function is the foundation of numerous diseases (56). The protection of intestinal function can be regulated by SCFAs. By activating 5’-adenosine monophosphate-activated protein kinase (AMPK) and the TLR4 pathway, butyrate in SCFAs may enhance the defensive function of intestinal epithelial cells by promoting mucin secretion (57) (**Figure 2A**). Additionally, sodium butyrate, a short-chain fatty acid, has been shown to increase the transcription of Claudin-1 by promoting binding between SP1 and the Claudin-1 promoter region (55). This enhancement of intestinal barrier function aids in maintaining overall intestinal homeostasis, thereby preventing the onset and development of tumors. Polyphenol metabolites are a type of microbial metabolite in the gut that help prevent CRC by modifying phytanic acid synthesis, downregulating inflammatory cascades, regulating DNA synthesis, and inducing luminal detoxifying enzymes (58). These actions aid in DNA repair, inhibit colonic pathogens, and regulate apoptosis, thus supporting the prevention of CRC (59). Quercetin-related metabolite indole-3-propionic acid acts as an AhR agonist. It suppresses inflammatory responses in colonic epithelial cells, thereby inhibiting carcinogenesis and exhibiting anticancer effects (60). Conjugated linoleic acid has the ability to inhibit DNA synthesis and induce apoptosis in human colon adenocarcinoma cell lines, and subsequently to alter the cell cycle of colon cancer cells, and consequently to reduce the incidence of colon cancer (61). Lactocin, a bacteriocin synthesized by Lactobacillus, has been extensively investigated in numerous studies for its anti-cancer effects on cell proliferation. It has been shown to increase the Bax/Bcl-2 ratio, an indicator of increased apoptosis index, and to promote apoptosis in colorectal cancer cells (62–64) In summary, the gut microbiota generates a variety of metabolites that exhibit anti-tumor activity through the inhibition of tumor cell proliferation, the regulation of tumor cell apoptosis, and the suppression of inflammatory responses. It is noteworthy that butyrate, as the most extensively studied SCFA, has been proven to have a dual role, with the ability to both promote tumorigenesis and produce anti-tumor effects through pathways such as maintaining intestinal barrier function.

### 3.2 The anti-oncogenic effect of digestive tract microbiome through modulation of the immune response

Bacteria exhibit anti-tumor immune responses through their ability for targeted, specific colonization of tumors and their immunogenic properties (65). The immunogenic capabilities of bacteria primarily manifest in their components, including peptides, polysaccharides, lipopolysaccharides, lipoteichoic acids, flagella, DNA, RNA, and others, which can be recognized by pattern recognition receptors on dendritic cells (DCs), macrophages, and neutrophils. This recognition subsequently triggers the appropriate immune responses, activating both the innate and adaptive immune systems, thus generating an anti-tumor effect (66, 67). To be specific, by increasing the expression of tumor necrosis factor-α (TNF-α) and IL-1β in the TME, the LPS component of Salmonella can enhance the functionality of immune cells, including CD8^+^ T cells, and generate an anti-tumor immune response (**Figure 2B**). Chen et al. have demonstrated that Salmonella flagella activate immune cells via the TLR5 signaling pathway, triggering a host immune response and resulting in a therapeutic effect against melanoma (68). Moreover, PAMPs expressed by Listeria infecting tumor tissues can be recognized by Toll-like receptors on antigen-presenting cells. This recognition activates the NF-κB pathway, subsequently clearing tumor cells (69). Attenuated Listeria has been used as antigen-presenting vectors, leading to the development of several malignant vaccines, including cervical cancer vaccines (70), with studies demonstrating efficacy. Additionally, Kuugbee’s research team investigated the effect of low-fructo-oligosaccharide maltodextrin-enriched Lactobacillus acidophilus, Bifidobacterium bifidum, and Bifidobacterium infantum (LBB) on the progression of CRC. They discovered that LBB boosts intestinal mucosal epithelial barrier integrity and decreases tumor incidence by promoting epithelial cell apoptosis and inflammation through the TLR2 pathway in the host (71).

### 3.3 The anti-oncogenic effect of bacterial outer membrane vesicles

Bacterial outer membrane vesicles (OMVs) are generated by gram-negative bacteria, composed by bacterial outer membrane component, and carry the essential antigenic components required to induce protective immune responses (72). They facilitate antigen presentation, activate the immune system, and generate anti-tumor effects. OMVs contain numerous PAMPs, capable of activating adaptive immune responses, generating antigen-presenting cells and interacting with pattern recognition receptors, ultimately activating antigen-presenting cells (73), leading to inhibitory effects on tumors. Kim et al. discovered that OMVs had the potential to target tumor issues and accelerate in tumor cells, following by inducing the production of anti-tumor factor CXCL10 and γ interferon (IFN-γ), as well as activating human immune response. This stimulation of the immune response leads to the inhibition of existing tumors and also inhibits tumor metastasis (74) (**Figure 2C**). Also, OMVs can produce intrinsic anti-tumor effects by delivering various toxic factors. Additionally, OMVs can cause the extravasation of red blood cells in the tumor area, leading to the aggregation of hemoglobin within the tumor. This results in a noticeable darkening of the tumor’s color and an increase in absorbance in the near-infrared region. Near-infrared lasers can be utilized to eliminate tumor cells (75). OMVs, as a promising means of treating tumors, can be enhanced in their anti-cancer effects through engineering modifications or their use as a drug carrier.

### 3.4 The anti-oncogenic effect of bacterial toxins

Bacterial toxins, synthesized during the metabolism of bacteria, are the material that have apparent toxic function to human body. They can form channel on the cell membrane of eukaryotic cells, thereby disrupting its barring function (**Figure 2D**), resulting in the anti-tumor effect (76). Research indicates that Salmonella typhimurium or *E. coli* can produce bacterial toxins, making their inhibitory effects on tumor onset and development more pronounced (77, 78). Additionally, Jiang and collogues proved that a cytolysin A produced by *Salmonella typhimurium*, *E. coli*, and *Shigella flexneri* can exert anti-tumor effects through two pathways: direct killing of tumor cells and promotion of tumor cell apoptosis by releasing membranous vesicles (78). Wang et al. indicated that Salmonella carrying the cell-killing expansin toxin B could inhibit tumor growth and extend survival by inducing apoptosis in tumor cells (79).

### 3.5 The anti-oncogenic effect of digestive tract microbiome through inhibition of tumor metastasis

Tumor metastasis is one of the main causes of the death of cancer patients, involving the capability of transference from primary tumors to other parts of the body. Conversely, bacteria, as organism, can affect the physiological functions of host cells, thus have the potential to inhibit tumor metastasis. Zheng et al. has proposed a method for using Salmonella to treat tumors, wherein the expression of Vibrio vulnificus flagellin B by attenuated Salmonella can reduce immune suppression, alleviate subcutaneous colorectal tumors in mice, and inhibit tumor growth and metastasis, leading to an extended lifespan (80). In addition, research proved that Listeria infection can activate nicotinamide adenine dinucleotide phosphate and increase the intracellular Ca^2+^ content (81), leading to the direct killing of tumor cells. Both mechanisms can generate a highly cytotoxic free radical - reactive oxygen species (ROS) (**Figure 2E**) (82). ROS can initiate immunogenic cell death in tumor cells, subsequently activating CD8 T cells to eliminate residual tumor cells, ultimately inhibiting tumor cell metastasis (83).

### 3.6 Others

Apoptosis is a cell-autonomous, orderly mode of cell death that occurs in nucleated cells under genetic regulation. Research by Hiroaki and colleagues has demonstrated that iron-siderophores in Lactobacillus could induce apoptosis through the Jun N-terminal kinase pathway, thereby inhibiting the growth of CRC (84). Yaser et al. manifested Bifidobacterium infantis had the ability to activate p53 and inhibit NF-κB, thus inducing apoptosis in colorectal cancer cells (85). Furthermore, angiogenesis provides malignant tumors with a rich nutrient supply, further promoting their development. Therefore, inhibiting angiogenesis is a direction for treating malignant tumors. By inhibiting angiogenesis, downregulating inflammatory responses, bacteria such as Lactobacillus and Bifidobacterium can therefore preventing CRC and reducing carcinogenic metabolites like SCFAs, ultimately enhancing the function of the intestinal barrier (86). In summary, digestive tract bacteria can play a role in treating tumors through immunomodulatory effects, the secretion of bacterial outer membrane vesicles, the production of bacterial toxins during metabolism, inducing apoptosis in tumor cells, and inhibiting tumor angiogenesis.

## 4 Natural bacterial-based tumor therapies

Digestive tract microbiome can be divided into three categories based on its function to human body. The first class comprises commensal bacteria, such as Lactobacillus and Bifidobacterium, which have a positive impact on human health and also hold potential for tumor therapy, often referred to as “anti-tumor bacteria.” The purpose of treating tumor can be achieved by increasing the amount of these “anti-tumor bacteria.” The second category is conditionally pathogenic bacteria, like *E. coli*, which, under normal circumstances, do not produce adverse effects on the host organism but can become harmful when the host’s immune defenses are compromised. Finally, pathogenic bacteria are the last class, involving Staphylococcus aureus, which have detrimental effects on the host’s health, including the potential to induce inflammatory responses and facilitate tumor initiation and progression, earning them the title of “pro-tumor bacteria.” Furthermore, Helicobacter pylori (*H. pylori*) is a bacterium that causes inflammation of the stomach lining, which can lead to stomach ulcers. Untreated, it can be a lifelong infection or a predisposition to stomach cancer (87). Consequently, the modulation of the gut microbiota has emerged as a novel approach for addressing diseases associated with gastrointestinal dysbiosis. Potential strategies for targeting the gut microbiota encompass the use of probiotics, fecal microbiota transplantation (FMT), the administration of anti-tumor antibiotics, and dietary interventions, among others.

### 4.1 Treating tumors by increasing the levels of “anti-tumor bacteria” within the body

Probiotics beneficial to the human body mainly include yeast, probiotic spores, lactobacillus, bifidobacterium, and actinomycetes (88). With the advancement of modern research, the study of probiotics has become more and more extensive. Research has demonstrated that probiotics can colonized in the intestinal of human body and promote overall health, through regulating the body’s immune response or modulating the balance of GIT microbiome, thereby maintaining intestinal homeostasis, among other mechanisms (89) (as shown in **Table 1**). Extensive studies retrieved by Stephanie et al. indicate that probiotics play a crucial role in preventing the development of colon cancer through mechanisms such as enhancing the function of intestinal barriers, suppressing and preventing colorectal carcinogenesis, reducing the metabolism of carcinogens, and inhibiting the growth of pathogenic bacteria (96). Consequently, probiotics have proven to be crucial in the field of cancer therapy.

**Table 1:** Representative probiotics and their tumor control mechanisms.

Probiotics strain(“anti-tumor bacteria”)	Types of tumor	Mechanisms that produce therapeutic oncological effects	Ref
Lactobacillus and Bifidobacterium mixture	Gastrointestinal tumors	Associated with improved intestinal integrity, TLR2-mediated cellular pathways, reduced size of gastrointestinal tumors and reduced tumor incidence	(90)
Lactobacillus casei BL23	Colon Cancer	Promoting the production of a balanced adjuvant Th-17 biased immune response	(91)
Pediococcus pentosaceus GS4	Colon Cancer	Reduces chronic inflammation and decreases NF-κB activity associated with cell proliferation	(92)
Lactobacillus and Bifidobacterium	CRC	Reduces the size and incidence of tumors	(71)
Probiotics mixture	liver cancer	Reduces the level of Th 17 cells in the gut and the extent of Th17 recruitment at the tumor site, changes the composition of the gut microbiota and reduces the size of liver tumors	(93)
Lactobacillus acidophilus	Breast cancer	Promotes the production of IFN-γ and reduces the production of IL-4, thereby enhancing the body’s immune response and producing an anti-breast cancer effect	(90)
Lactococcus lactis NK34	Breast and lung cancer	Inhibits proliferation of tumor cells	(94)
Bifidobacterium	Melanoma	Reduces tumor volume of Melanoma	(95)

**Figure 3:**
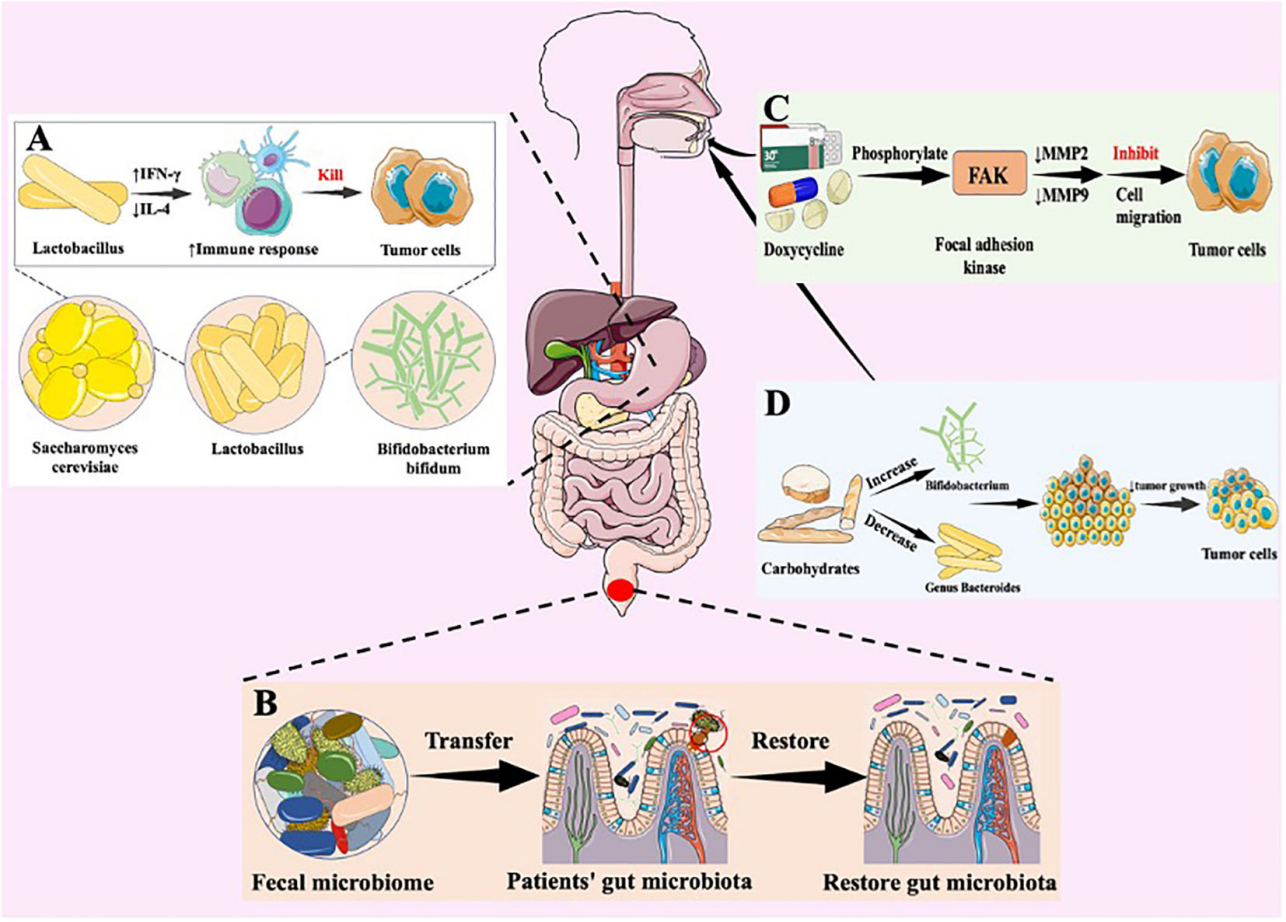
Natural bacterial-based tumor therapies. Various strategies designed to target the gut microbiota and modulate its composition for potential therapeutic benefits. **(A)** Antitumor mechanism of probiotics. Lactobacillus acidophilus is a probiotic that boosts the immune response through an increase in interferon-gamma (IFN-γ) production and a decrease in interleukin-4 (IL-4) levels. This balance leads to an increase in Th1 responses, which is critical for robust anti-tumour activity. Stimulating IFN-γ helps activate immune cells to target and destroy cancer cells, including breast cancer cells, thereby boosting the body’s anti-tumour defences. **(B)** therapeutic effect of fecal microbiota transplantation. FMT symbolized by the transfer of healthy fecal material, aims to enhance microbial diversity and establish a healthy microbial community within the gut environment. FMT are introduced into the gut to restore microbial balance and promote a symbiotic relationship. **(C)** Antitumor mechanism of anti-tumor antibiotics. Doxycycline (DOX) inhibits leukaemia cell migration through phosphorylation of FAK and reduction of MMP-2 and MMP-9 expression. These enzymes are involved in the degradation of the extracellular matrix, which aids cell migration. DOX therefore reduces the ability of leukaemia cells to move and spread. **(D)** Dietary interventions. These interventions selectively nourish beneficial bacteria and contribute to the modulation of microbial composition.

In phase I clinical trials, influence of probiotics on gastrointestinal tumors have primarily been focused on (90, 97). *In vitro* experiments suggest that the inhibitory effect of probiotics on gastrointestinal tumors primarily relies on the production of SCFAs by probiotics, which subsequently (97). Additionally, probiotics also exhibit inhibitory effects on extraintestinal tumors such as liver cancer, breast cancer, lung cancer, and melanoma (71, 90–95). Imani and colleagues have confirmed that oral administration of Lactobacillus acidophilus, a type of probiotic, can stimulate the generation of IFN-γ while reducing the production of IL-4, thereby enhancing the immune response, inhibiting breast cancer cells, and strengthening the anti-tumor effect (**Figure 3A**) (90). Furthermore, probiotics also demonstrate inhibitory effects on extraintestinal tumors. Jun Li et al. have developed a novel probiotic mixture, which can slow down tumor growth and reduce tumor volume and size (93). In conclusion, probiotics hold great promise and untapped potential in the treatment of cancer, providing new research directions in the field.

FMT involves extracting microbial communities from healthy individuals’ feces that are effective against a specific disease and transplanting them into patients to restore the patients’ gut microbiota, thereby achieving therapeutic effects (**Figure 3B**) (98, 99). Rosshart et al. demonstrated that laboratory mice transplanted with gut microbiota from wild mice exhibited better resistance to CRC than control mice with their native bacteria (100). Meanwhile, gut bacterial metabolites have been shown to promote the development of chronic liver disease and hepatocellular carcinoma through the gut-liver axis (101). Recent research strongly supports the role of FMT in controlling the progression of liver cancer, such as its potential to prevent alcohol-induced liver injury (102). Moreover, FMT holds promise for enhancing the anti-tumor immune response in melanoma patients by transferring a beneficial gut microbiota community (101). Gopalakrishnan and colleagues transferred fecal samples from melanoma patients to mice and observed that FMT could enhance the effectiveness of tumor immunotherapy (103).

### 4.2 Treating tumors by decreasing the levels of “pro-tumor bacteria” within the body

In the human digestive system, in addition to probiotic colonization, numerous harmful bacteria can trigger inflammatory responses that may lead to the onset and development of tumors. Therefore, the use of antibiotics to inhibit or eliminate these bacteria have been proved to provide therapeutic effect on tumor. Antibiotics have the capability to exhibit cytotoxicity against tumor cells and can exert inhibitory effects on tumors through various mechanisms. Doxycycline (DOX) have been proved possess cytotoxicity and anti-proliferative properties against various cancer cells (104, 105). Moreover, DOX exhibits broad therapeutic characteristics, such as controlling invasive and metastatic cancer cells, including inhibiting tumor growth and suppressing tumor cell migration (106). DOX inhibits leukemia cell migration by phosphorylating focal adhesion kinase, resulting in decreased expression of matrix metalloproteinases MMP2 and MMP9, which contribute to cell migration (**Figure 3C**) (106, 107). Yang and colleagues have demonstrated that DOX induces apoptosis in cervical cancer cell lines, inhibits tumor cell invasion, and reduces cancer stem cell (CSC) markers in cell culture, such as SOX-2 and OCT-4 (108, 109). Also, salinomycin (SAL), isolated from Streptomyces albus, has been shown not only to inhibit the proliferation of various tumor cells but also to suppress multidrug resistance (110, 111). Simultaneously, SAL is capable of targeting multiple malignant tumor CSCs while enhancing the effectiveness of radiotherapy and chemotherapy. The application of antibiotics can eliminate numerous bacteria in the gastrointestinal tract, ultimately decreasing the toxic adverse consequences that arise after cancer treatment.

### 4.3 Treating tumors through well-balanced diet

A well-balanced diet is essential in delaying the onset and progression of tumors. Therefore, it is necessary to follow a diet that includes a wide range of food items to prevent the growth and spread of tumors. Consuming various foods not only alters the composition of the gut microbiota but also impacts the relative changes in gut metabolites (11). For example, the intake of carbohydrates can increase the relative abundance of Bifidobacterium in the gut, while decreasing the abundance of the genus Bacteroides (112). Also, Bifidobacterium belongs to the group of probiotics and has been associated with potential benefits in preventing inflammatory bowel diseases and CRC (**Figure 3D**) (113). Mehta et al. have illustrated that consuming grains and foods rich in dietary fiber may increase the likelihood of CRC FN infection (114). In addition, in patients with chronic liver disease, including those with hepatocellular carcinoma, consumption of fermented dairy products has a therapeutic effect in reducing symptoms of abdominal distension (112). Intake of probiotics enables the increase of diversity of *in vivo* microbiome, thus improving anti-tumor immune response (11). Consequently, a well-balanced diet is crucial in regulating the composition of GIT microbiome, thereby to some extent beneficial to the treatment of tumors.

## 5 Strategies for artificially modifying bacteria to treat tumors

The unique TME renders normal tissue blood vessels inadequate to support tumor growth during its sustained proliferation and expansion. Consequently, tumor cell growth factors activate to generate novel vascular structures (115). Such disordered vascular structure and TME characteristics present several constraints to current tumor treatment methods. These include the challenge of chemotherapeutic drugs penetrating tumor cells and their proneness to off-target effects (116). Conversely, the complexity of tumor vascular structure not only enables bacteria been trapped inside the tumor, but also provide nutrients to it, contributes to promoting the proliferation of bacteria. Consequently, bacteria can undergo artificial modification, including using engineered bacteria or bacterially incorporated nanomaterials, to enhance safety and targeting, resulting in improved anti-tumor effects.

### 5.1 Engineered bacteria

Genetic engineering and biological synthesis are employed to modify bacteria, aiming to achieve tumor targeting and reduce toxicity. This emerging strategy boasts numerous advantages, including a considerable enhancement in safety and specificity, as well as the inhibition of angiogenesis for anti-tumor effects, and the ability to interfere with tumor growth through RNA interference (117). Yu and colleagues employed a unique synthetic biology technique to reprogram Salmonella typhimurium to survive solely under anaerobic conditions, retaining its functionality. The team experimentally confirmed its ability to hinder tumor growth in mice, while leaving healthy cells unharmed. The engineered bacterium outperformed standard Salmonella typhimurium by reducing its toxicity and exhibiting anti-tumor properties (118). Simultaneously, engineered bacteria can be designed to target the increased angiogenesis characteristic of solid tumors and inhibit their growth, thereby offering therapeutic benefits. Jia et al. utilized genetic modifications to create attenuated Salmonella typhimurium, which delivered STAT3-specific siRNA and endostatin to mice with liver cancer. Their combination therapy demonstrated a more significant effect in treating tumors (119). As a result, engineered bacteria, as an incredibly promising method for treating tumors, are currently the subject of extensive research.

### 5.2 Bacteria combined with nanomaterials to exert anti-tumor effects

**Figure 4:**
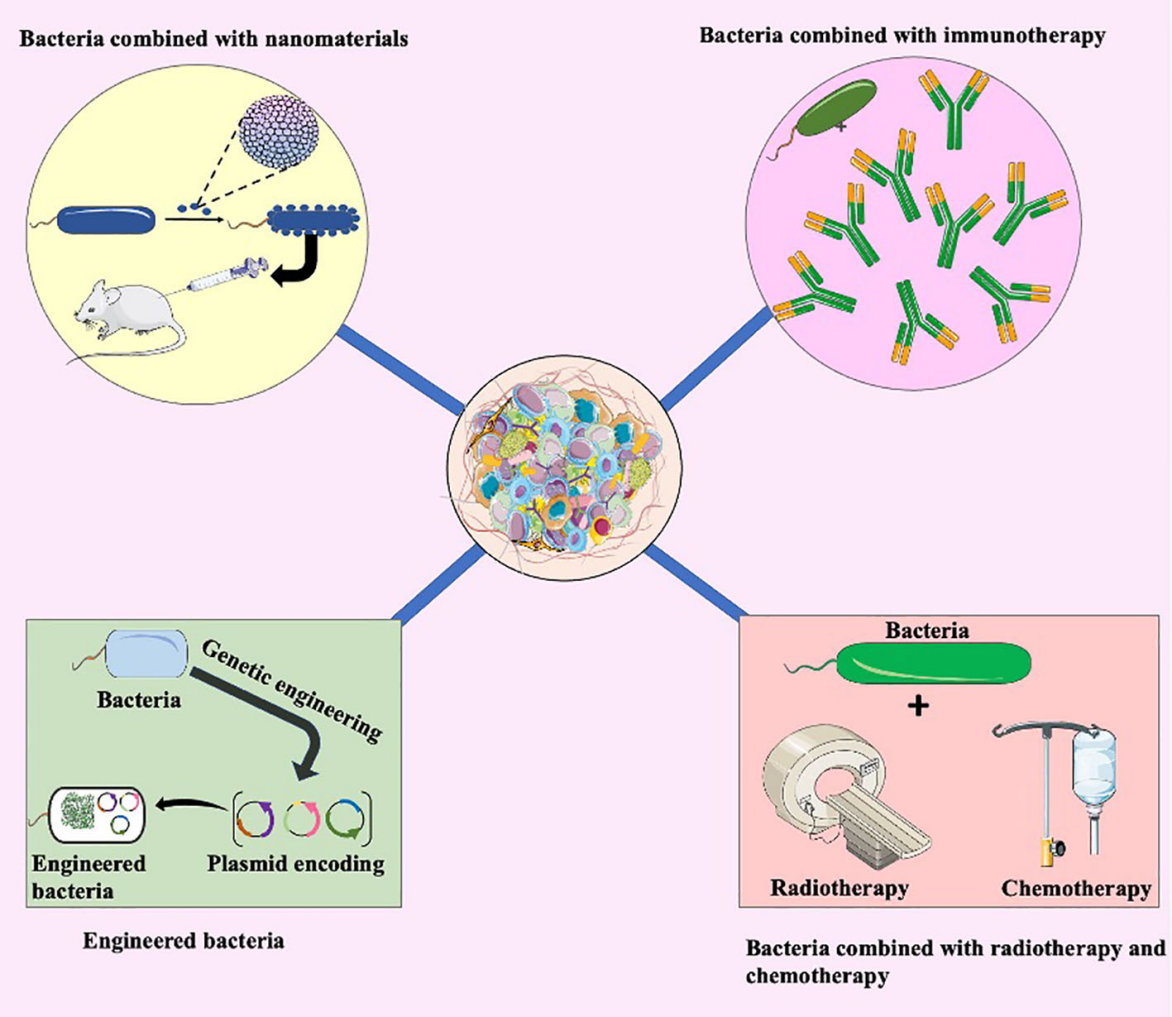
Bacterial combination therapy. The integration of bacteria with nanomaterials represents an innovative approach. Nanomaterials enhance the delivery of therapeutic agents, optimizing their interaction with tumors. This synergy holds potential for more targeted and effective tumor treatment. Bacteria combined with immunotherapy illustrates another promising avenue. The interaction between bacteria and the immune system can be harnessed to enhance the body’s natural defense mechanisms against cancer cells. This combination approach aims to amplify the immune response for improved tumor recognition and elimination. Engineered bacteria involve genetic modifications to enhance their therapeutic properties. These modifications may include the production of anti-tumor agents or the expression of specific receptors for targeted delivery within the tumor microenvironment. Additionally, the integration of bacteria with traditional cancer treatments such as radiotherapy and chemotherapy. Bacteria can potentially augment the efficacy of these treatments, creating a synergistic effect for more potent tumor eradication.

Nanomaterials’ unique high penetrance and retention effect of nanomaterials enable them to accumulate at the tumor site, enhancing their targeting capabilities. Therefore, they find wide applications in cancer therapy (120). Currently, research indicates that bacteria can be chemically attached to nanomaterials, offering a straightforward and efficient technique. Moreover, Liu et al. have proposed innovative binding methods. To be specific, Quantum dots, due to their remarkable photostability and luminescence traits, demonstrate considerable promise in bioimaging and diagnostic applications (121). Then, Liu’s team utilized bacteria as carriers to deliver quantum dots specifically into solid tumors, introducing a novel method of combining bacteria and nanomaterials (121). In order to achieve precise tumor therapy, Chen and colleagues associated Salmonella enterica YB1 to nano-photosensitizers by loading indocyanine green nanoparticles through an amide bond (122). Zhang’s team devised a technique that entails loading composite nanoparticles of gold and platinum (Bac-Au@Pt) onto bacteria’s surface to overcome the hindrance of antioxidant effects by tumors during chemodynamic therapy, as well as to decrease negative consequences on neighboring cells (123). **Figure 4** provides an overview of the approaches to treating tumors through the bacteria’s collective action.

### 5.3 Bacteria in combination with conventional therapies for treating tumors

Currently, radiation therapy, chemotherapy and immunotherapy are the main non-surgical approaches to tumor treatment. However, these methods lack malignancy specificity and often cause adverse effects (78, 124). By contrast, if combine bacterial treatment to conventional therapies can overcome the disadvantages like poor targeting and specificity of conventional therapies. **Table 2** summarizes part of research progress associated with the combination of bacterial treatment and conventional therapies.

**Table 2:** Bacteria combined with traditional methods to treat tumors.

No.	Traditional oncology therapy in combination with bacteria	Name of bacteria	Drug	Types of tumor	Mechanism of action	Ref
1	Radiotherapy	*Salmonella*		systemic antitumor effects	Salmonella activates peripheral DCs in tumor marginal tissues, resulting in increased DC numbers and prolonged survival to produce good tumor therapeutic effects.	(125)
2	Radiotherapy	*E. coli*		CRC	Significant reduction in tumor size and production of cytolysin A	(78)
3	Chemotherapy	*E. coli* Nissle 1917 (EcN)	doxorubicin (DOX)	Breast cancer	Adriamycin is coupled to EcN via the acid instability of cis-aconitic anhydride and this coupling not only increases its viability in tumor tissue but also has no effect on bacterial motility	(126)
4	Chemotherapy	*Salmonella*	doxorubicin	CRC	With the help of high intensity focused ultrasound heating, the release of adriamycin from colon cancer cells is induced, thus allowing the drug to take effect in the cytoplasm and nucleus of the cancer cells.	(127)
5	Immunotheraoy	Listeria	gemcitabine	pancreatic ductal adenocarcinoma	Selectively targets tumors and can be delivered to the tumor area to act	(128)
6	Immunotherapy	*L. acidophilus*		Lung cancer	Increase in serum levels of IFN-γ, IL-10, CD4 and CD8 cells	(129)
7	Immunotherapy	*L. monocytogenes*		Melanoma	Enhanced infiltration of CD4 and CD8^+^ T cells	(130)

Radiotherapy is a focused cancer treatment using radiation to eradicate cancer cells. Its principle is to target the tumor and avoid damage to healthy adjacent cells. Combining bacteria with radiation therapy for cancer treatment can enhance the targeting of bacteria to cancer cells, prolong the duration of bacterial action, and improve the overall effectiveness of radiation therapy. This approach holds promise in improving the outcomes of cancer treatment (131). Chandra and colleagues utilized a starvation response to introduce the radioactive isotope (32) P into Listeria bacteria. This allowed for the induction of ionizing radiation and bacteria-induced reactive oxygen species at the tumor site, ultimately resulting in the targeted eradication of cancer cells (132).

Chemotherapy drugs can spread throughout the body via the bloodstream and are a primary treatment method for inhibiting metastatic cancer (133). The disruption of tumor-specific vascular structures by chemotherapeutic agents can be enhanced by using bacteria in combination with chemotherapy for cancer treatment. Studies indicate that a combination of Salmonella VNP20009 with cyclophosphamide significantly reduces tumor micro-vessel density and vascular endothelial growth factor content compared to using cyclophosphamide alone (134). Meanwhile, numerous studies suggest that bacteria can act as chemotherapy drug carriers, facilitating targeted cancer therapy and reducing drug side effects (135).

Immunotherapy is the method that enhancing the immune system of immunocompromised patients. Recent research indicates that tumor cells have developed numerous mechanisms to evade immune detection, including recruiting immunosuppressive cell populations, modulating signaling pathways, and altering the TME, reducing immunotherapy efficacy (136). To be specific, tumor cells can evade host immune surveillance by expressing PD-L1 and activating PD-1 (137). whereas the microbiome is intimately involved in the regulation of checkpoint interactions, for example, bifidobacterium bifidum in combination with anti-PD-L1 therapy improves efficacy through activation of dendritic cells and enhanced accumulation of CD8^+^ T cells (95). In addition, immune checkpoint inhibition is the key to anti-cancer immunotherapy, which enhances the immune response by blocking immune checkpoints such as CTLA-4 and PD-1 (138, 139), such as Ipilimumab. The monoclonal antibody is currently used in the treatment of melanoma, renal cell carcinoma, hepatocellular carcinoma, non-small cell lung cancer and colorectal cancer. Bacteria, genetically engineered to increase the expression of tumor antigens, can be combined with immunotherapy to boost the body’s immune response, resulting in an anti-tumor response.

### 5.4 Others

Photothermal therapy (PTT) and photodynamic therapy (PDT) have a huge potential to become a vital method to treat tumors (140). However, the mechanism of such two treatments not fully understood yet, making it difficult to achieve optimal treatment results. At the same time, these two methods all approaches confront challenges such as limited penetration depth and the suboptimal tumor-targeting capabilities of photosensitizers. However, obligate or facultative anaerobic bacteria are naturally endowed with specific targeting capabilities for hypoxic regions within the tumor. Furthermore, the increased permeability of tumors enables enhanced binding between internal chemotactic factors and bacterial-specific receptors, promoting bacterial colonization within the tumor. Once established, bacteria can inhibit tumor growth through mechanisms such as immunomodulation (141). Due to the innate targeting capabilities of bacteria towards tumor cells, bacteria can be conjugated with photosensitizers to facilitate the delivery of these photosensitizers to the tumor site (122). Simultaneously, bacteria activate the host immune response, thereby significantly improving the effectiveness of PDT and PTT in treating tumors.

### 5.5 Clinical research based on bacterial tumor treatment

Bacterial-based tumor therapy has plenty of advantages compared to conventional drug delivery systems. Firstly, bacteria such as Bifidobacterium, Salmonella, Escherichia and Clostridium can selectively target and proliferate within tumour tissue due to their innate ability to thrive in the hypoxic and acidic environments characteristic of solid tumours. These bacteria can penetrate the blood-tumour barrier and deliver therapeutic agents directly to the tumor site by exploiting the unique conditions of the tumour microenvironment. In addition, the anti-tumor effects of bacteria can be enhanced by their inherent immunogenicity and toxin production. Recent advances in genetic engineering have reignited interest in bacterial therapy as a promising modality for cancer treatment by further optimising these bacterial vectors, enabling precise targeting and minimising off-target effects. Based on the huge progress in laboratory research of bacterial tumor treatment, numerous clinical research has also been undertaken (**Table 3**).

**Table 3:** Clinical trials related to the treatment of tumors with bacteria.

No.	Company	Bacteria	Indications	Clinical Phase	Clinical Trials No.	Ref
1	Jianbin Xiang, Huashan Hospital	intestinal maladjusted flora	Rectal Cancer	Early Phase 1	NCT05759741	(142)
2	Mayo Clinic	Engineering Gut Microbiome	Breast and Lung Cancer	Early Phase 1	NCT04857697	(143)
3	National Cancer Institute (NCI)	gut microbiome	melanoma	Phase I Phase II	NCT03819296	(144)
4	Parker Institute for Cancer Immunotherapy	oral microbiome	Metastatic Melanoma	Phase 1	NCT03817125	(145)
5	Shanghai Zhongshan Hospital	intestinal flora	Non-Small Cell Lung Cancer	Phase 1	NCT05008861	(146)
6	Michael Dill, University Hospital Heidelberg	Fecal Microbiota	hepatocellular carcinoma	Phase 2	NCT05690048	(147)
7	National Institutes of Health Clinical Center (CC) (National Cancer Institute (NCI))	gut bacteria	Metastatic Hepatocellular Carcinoma	Phase 2	NCT04025567	(148)
8	National Institutes of Health Clinical Center (CC) (National Cancer Institute (NCI))	gut bacteria	Hepatocellular Carcinoma	Phase 2	NCT03785210	(149)

## 6 Future perspective

The gastrointestinal tract encompasses the human digestive process, from ingestion to excretion, and contains a diverse array of bacteria. The gut microbiota has been implicated in several gastrointestinal diseases. The connection between the gut microbiota and the development and progression of tumors is intricate, with different gastrointestinal bacteria exerting varying influences on tumorigenesis and growth. Some bacteria have potential in tumor therapy because they promote tumorigenesis, while others inhibit tumor cell growth. Apart from their intrinsic anti-tumor effects, bacteria can be genetically engineered to achieve specific targeting. Bacteria targeting tumors can overcome the unique tumor microenvironment and overcome the limitations of current clinical methods of treating tumors, which fail to penetrate deep into the tumor. Accordingly, bacteria hold great promise as a modality for the treatment of tumors. More complex goals in tumor therapy can be achieved by combining bacteria with lightweight nanomaterials. Furthermore, bacterial combination therapy is a prominent area of research seeking to overcome the limitations of current tumor treatment methods. Presently, several clinical trials related to bacterial therapy of tumors have entered either phase I or phase II. Consequently, bacterial therapy for tumors has the potential to become a novel frontier in future tumor treatment.

## Author contributions

YS: Writing – original draft, Writing – review & editing. XL: Writing – review & editing, Funding acquisition. JZ: Writing – review & editing, Funding acquisition.
